# The Fetal Modified Myocardial Performance Index: Is Automation the Future?

**DOI:** 10.1155/2015/215910

**Published:** 2015-06-22

**Authors:** Priya Maheshwari, Amanda Henry, Alec W. Welsh

**Affiliations:** ^1^Faculty of Medicine, School of Women's & Children's Health, University of New South Wales, Sydney, NSW 2052, Australia; ^2^Department of Maternal-Fetal Medicine, Royal Hospital for Women, Locked Bag 2000, Barker Street, Randwick, NSW 2031, Australia; ^3^Women's and Children's Health, St George Hospital, Kogarah, NSW 2217, Australia; ^4^Australian Centre for Perinatal Science, University of New South Wales, Sydney, NSW 2052, Australia

## Abstract

The fetal modified myocardial performance index (Mod-MPI) is a noninvasive, pulsed-wave Doppler-derived measure of global myocardial function. This review assesses the progress in technical refinements of its measurement and the potential for automation to be the crucial next step. The Mod-MPI is a ratio of isovolumetric to ejection time cardiac time intervals, and the potential for the left ventricular Mod-MPI as a tool to clinically assess fetal cardiac function is well-established. However, there are wide variations in published reference ranges, as (1) a standardised method of selecting cardiac time intervals used in Mod-MPI calculation has not been established; (2) cardiac time interval measurement currently requires manual, inherently subjective placement of callipers on Doppler ultrasound waveforms; and (3) ultrasound machine settings and ultrasound system type have been found to affect Mod-MPI measurement. Collectively these factors create potential for significant inter- and intraobserver measurement variability. Automated measurement of the Mod-MPI may be the next key development which propels the Mod-MPI into routine clinical use. A novel automated system of Mod-MPI measurement is briefly presented and its implications for the future of the Mod-MPI in fetal cardiology are discussed.

## 1. Introduction

Fetal echocardiography has developed over the past 30 years as the primary noninvasive modality used to evaluate fetal cardiac anatomy, haemodynamics, and function [1]. Functional cardiac assessment using echocardiography is becoming increasingly valued because it facilitates the detection of subtle myocardial dysfunction during development and can guide management and improve neonatal outcomes [2]. Early intervention can prevent progression into intrauterine cardiac failure [3].

The myocardial performance index (MPI) is a noninvasive pulsed-wave Doppler-derived measure of global myocardial function which has been evaluated in fetal cardiology and is a ratio of isovolumetric to ejection time cardiac time intervals [1]. However, its clinical applications have been limited due to its poor reproducibility [4]. This review charts the development of this index as applied to the fetus and progress in technical refinements of its measurement, with particular focus on developments since the introduction and acceptance in 2005 of the modified-MPI, or Mod-MPI [5]. The Mod-MPI was defined using specific methodology for measuring the time periods required in fetal MPI calculation.

## 2. Development of the Pulsed-Wave Doppler Derived Myocardial Performance Index (MPI)

The MPI was first described for adult cardiac evaluation in cases of dilated cardiomyopathy in 1995 by Tei et al. [7]. Originally known as the Tei Index but later termed the MPI, it is traditionally a pulsed-wave derived index which incorporates measurement of cardiac time intervals and is defined as follows [8]:

(1) MPI=isovolumetric  contraction  time  ICTlllll+  isovolumetric  relaxation  time  (IRT) ×ejection  time  ET−1.

Both atrioventricular and ventricular ejection flows are evaluated to determine constituent time intervals. The ICT represents the time when myocardial contraction causes increased intraventricular pressure without an accompanying change in ventricular volume since all valves are closed during this phase [9]. The IRT refers to the postsystolic myocardial relaxation that occurs as calcium reuptake begins in cardiomyocytes, which decreases intraventricular pressure since no blood is entering or leaving the ventricles [9]. The ET starts when the ventricular pressure is raised sufficiently to open the aortic/pulmonary valves, causing the myocardium to deform and for blood to be ejected from the ventricle [10].

Myocardial dysfunction results in increased MPI values, predominantly due to a prolongation of the IRT although often accompanied by a reduced ET [1]. Calcium reuptake in cardiomyocytes is suppressed during cardiac functional deterioration which extends the time required for the myocardium to fully relax [11], and the IRT is the main MPI parameter to become abnormal early in cardiac dysfunction [10].

The MPI has been demonstrated to correlate well with other invasive and noninvasive measures of left ventricular function in adults [12]. Pulsed-wave Doppler-derived MPI was validated and became widely used as a quantitative measure of global cardiac function first in adults and then in the paediatric population [13–15]. The MPI is a particularly valuable index because precise anatomical imaging is not necessary in order to measure the MPI and it is independent of heart rate, blood pressure, and ventricular shape because the index only incorporates time intervals [7, 16, 17]. The fact that it is relatively easily measurable and can be incorporated into a routine ultrasound examination adds to its utility in the fetal context [18].

## 3. Application of the MPI to Fetal Cardiac Evaluation

Tsutsumi et al. in 1999 were the first to report the use of the MPI to evaluate global myocardial function in fetuses, using two waveforms and therefore two cardiac cycles [17]. Subsequent studies demonstrated a wide variability in normal reference values, likely contributed to by the lack of clear landmarks in the Doppler waveforms to calculate time intervals and the possible impacts of fetal heart rate variation between the two waveforms [7, 19, 20]. Measurement using two cardiac cycles also meant that individual measurements of the ICT and IRT could not be made.

**Figure 1:**
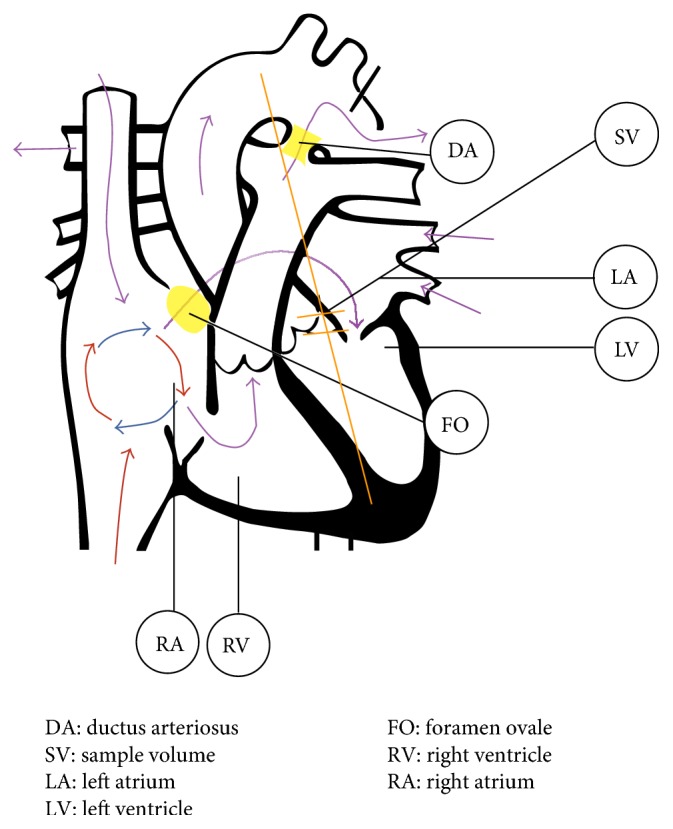
Positioning of the Doppler sample volume for acquisition of the left MPI. Modified from OpenStax College (2013) [6].

Friedman et al. proposed a new position for the Doppler sample volume in 2003 which enabled the left ventricular MPI to be evaluated from a single Doppler waveform [1]. Close proximity of the mitral inflow and aortic outflow tracts in the fetal heart allowed isovolumetric periods and ejection time to each be recorded simultaneously within the same cardiac cycle (see Figure 1).

The opening and closing of valve leaflets produces Doppler echoes or “clicks,” seen as vertical stripes on the Doppler waveform. The methodology for calculating the left MPI was further modified by Raboisson et al. in 2003 when they proposed that the Doppler click of the aortic valve opening be used as a landmark in order to better estimate the time intervals of MPI calculation [21].

**Figure 2:**
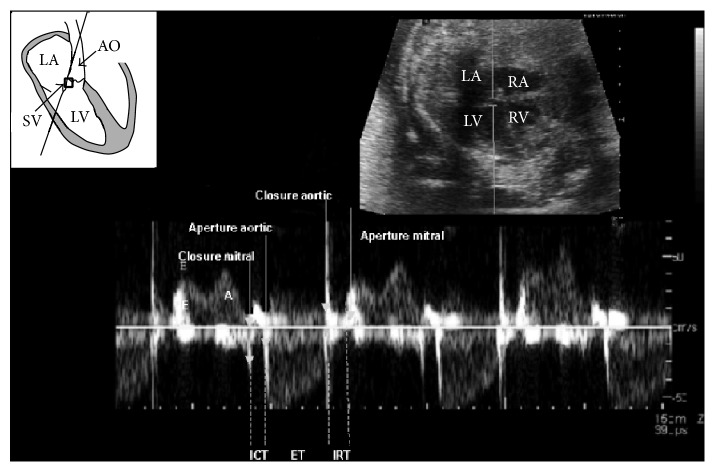
The schematic diagram at top left shows placement of the Doppler sample volume (SV) in left Mod-MPI measurement. The corresponding Doppler waveform is shown at bottom and the 2D echocardiograph is at top right. LV: left ventricle; LA: left atrium; RV: right ventricle; RA: right atrium; AO: aortic outflow tract; adapted from Hernandez-Andrade et al. (2005) [5].

**Figure 3:**
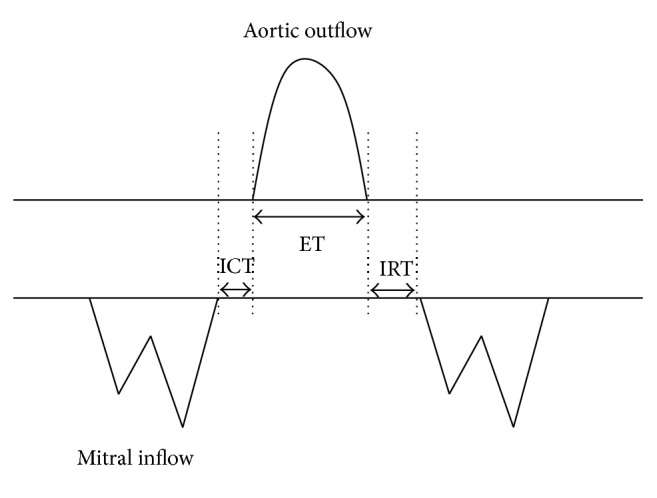
Time interval measurements are based upon the echoes from valve movements.

In 2005, Hernandez-Andrade et al. introduced the modified MPI (Mod-MPI) using the beginning of opening and closing Doppler clicks of both the aortic and mitral valves as measurement landmarks for determination of the different time periods (see Figures 2 and 3) [5]. This significantly reduced the inter- and intraobserver variability and thus improved reproducibility of the index in fetal medicine. For the left Mod-MPI, the Doppler sample gate was placed on the lateral wall of the ascending aorta close to the mitral valve in an apical four-chamber view of the fetal heart, as shown in Figures 1 and 2 [5].

The MPI has been used to demonstrate fetal cardiac dysfunction in a number of pathological conditions, including intrauterine growth restriction [17, 22–25], maternal diabetes [26–30], twin-twin transfusion syndrome (TTTS) [31–34], congenital heart malformations [35–39], preeclampsia [40], and other fetal conditions [41–44].

**Figure 4:**
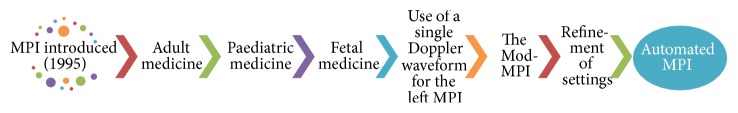
The main stages of evolution of the MPI since 1995.

Figure 4 summarises the developments leading to the Mod-MPI, and it also highlights the direction of research since then which will be analysed in the subsequent sections of this review.

## 4. Varied Measurements between Research Groups

Manual placement of a time calliper on the Doppler waveform is used to measure the time intervals between various clicks. Minor variations in calliper placement and hence alterations of mere milliseconds in time interval measurements result in significantly different Mod-MPIs [47]. For example, an alteration of only 4 milliseconds in constituent components of the MPI results in a variation in MPI of approximately ±12–14% [48].

**Figure 5:**
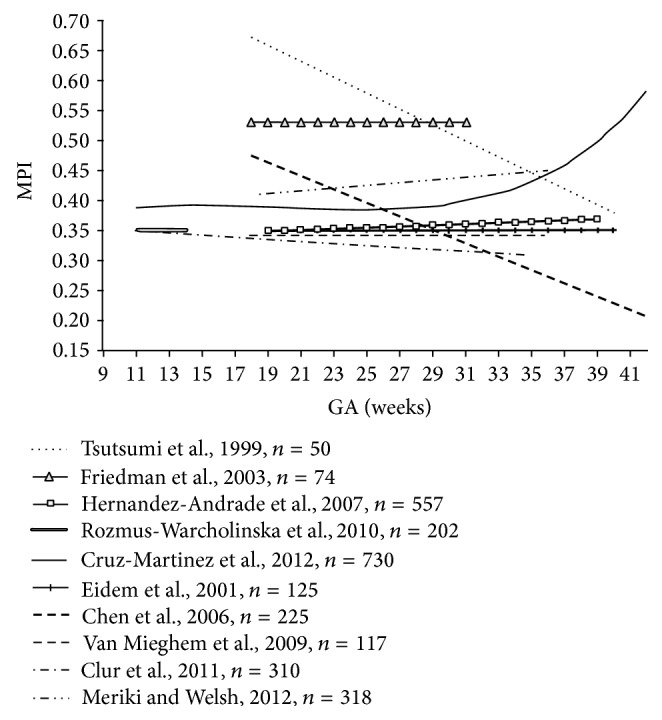
Gestational age-adjusted mean values for fetal left MPI in studies published from 1999 to 2012; adapted from Cruz-Martinez et al. (2012) [45, 46].

A lack of consensus on calliper placement between research groups has likely contributed to the wide range of values (0.35–0.60) for quoted “normal” Mod-MPI in singleton fetuses [1, 17–19, 45, 47, 49–52]. Some authors describe almost constant left MPI measurements throughout pregnancy, whilst others report gradual increases or decreases in mean MPI (Figure 5). These factors have restricted the translational potential of the MPI, as while individual research groups have demonstrated significant differences between their pathological subgroups and controls [24, 28, 49, 53], the lack of a universal reference range makes meaningful comparison of pathological findings between research groups impractical.

## 5. Technical Considerations

### 5.1. Calliper Placement

**Figure 6:**
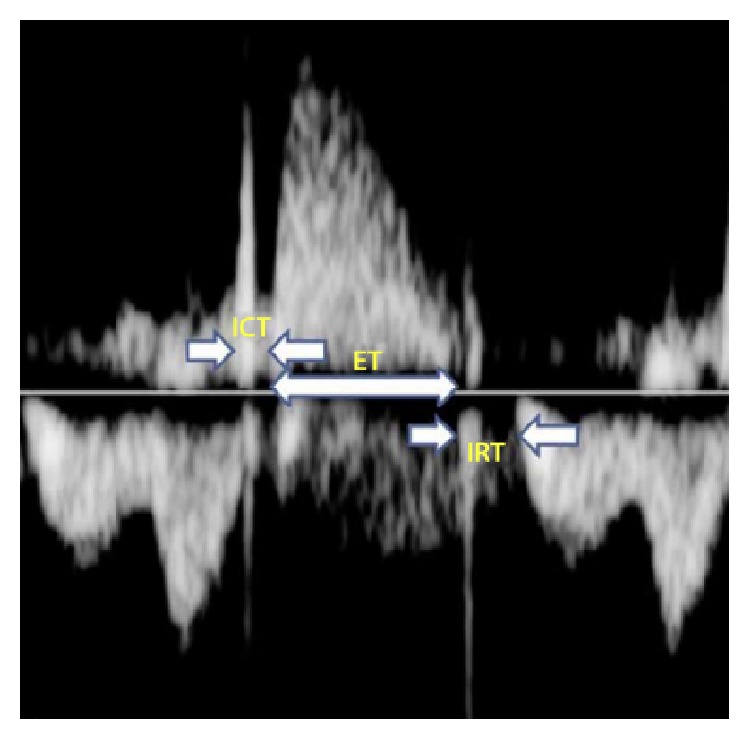
Left Mod-MPI Doppler waveform, showing the time intervals [47].

**Figure 7:**
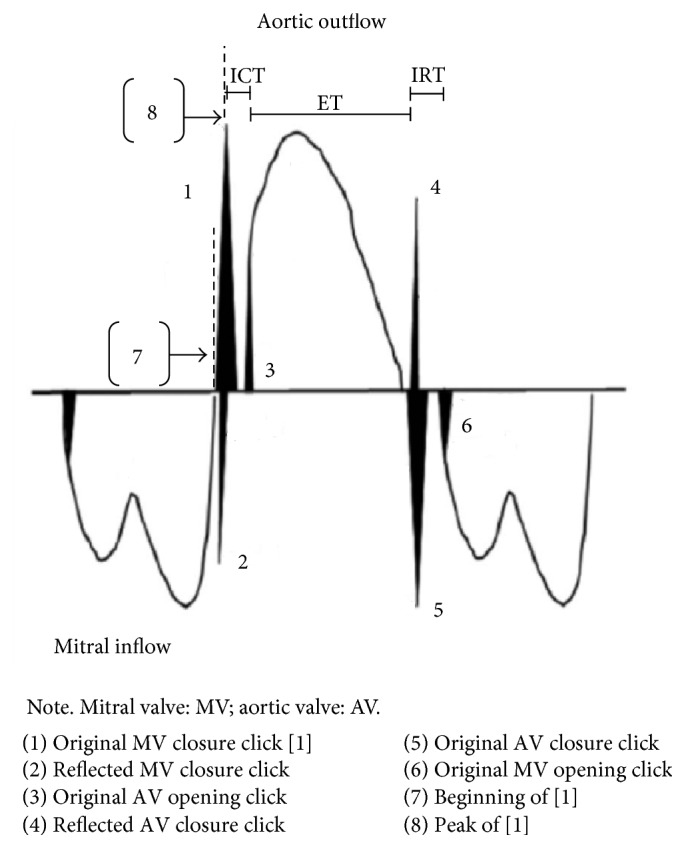
Schematic representation of valve clicks for the left Mod-MPI Doppler waveform, with a focus for time intervals on the original mitral valve closure click for simplicity; adapted from Meriki and Welsh [47].

The opening and closing of valve leaflets produces “original” Doppler clicks in the same direction as blood flow (for opening clicks) or opposite direction to flow (for closing clicks). Smaller Doppler echoes may be present in the opposite direction to the original clicks, termed “reflected” clicks (see Figures 6 and 7) [4]. The original and reflected clicks share a common peak time point, and it is suggested that thinner clicks enable more precise measurement [4].

Although the first normal range for fetal MPI was established using calliper positioning at the beginning (just before the echo) of the valve click [5], the criteria for demarcation of time intervals according to valve clicks are conflicting between subsequent studies. Some position callipers from the end of one click to the beginning of the next, corresponding to physiological time intervals as the period of valve movement is not included in ICT and IRT calculations [49]. A number of studies have failed to define a methodology for demarcating time intervals [28, 50, 51]. Other studies have positioned the calliper at the peak of valve clicks (see Figure 7) [47]. This is a clearer landmark that overcomes variations in valve click widths, though it does not absolutely correlate with the physiological time periods because the valves open at the start and close at the end of valve clicks. However, it has higher repeatability than the original method and it overcomes the limitations created by clicks of varying widths and therefore is likely to be a more useful method [54].

### 5.2. Machine Settings and Acquisition Technique

Hernandez-Andrade et al. described optimal settings as summarised in row 2 of Table 1 and suggested use of the fastest possible sweep velocity [55]. Higher sweep velocities create greater horizontal “stretch,” with clearer visualisation of valve clicks for more accurate time interval measurement. Low Doppler gain and a high-pass wall motion filter (WMF) were suggested in order to limit artefacts and noise and enable more precise recognition of clicks [55]. Meriki et al. further defined a fixed WMF at 300 Hz as providing improved repeatability and additionally stated that the angle of insonation should be kept less than 15° and Doppler aliasing avoided [48].

Lobmaier et al. have since investigated the impact on left Mod-MPI values of differing ultrasound settings (namely, sweep speed, gain, and WMF) and equipment [56]. They are the first to investigate the effect of using different equipment on Mod-MPI calculation and repeatability of measurement, specifically by comparing Mod-MPI values obtained from the Siemen Antares and Voluson 730 Expert ultrasound machines.

Lobmaier et al. concluded that raised sweep velocities and WMF resulted in superior measurement repeatability because of the association of those settings with higher intraclass correlation coefficients (ICCs), although optimal machine settings differ between manufacturers. Their results and the settings used by other groups are summarised in Table 1 [56]. In future research similar methodology needs to be applied to optimising MPI settings for different machines, which may result in different reference ranges depending on machine type used.

## 6. Automation of the MPI

The establishment of universal agreement for machine settings does not appear too distant if future studies can build on the findings of Lobmaier et al. [56]. Lack of a standardised method of selecting cardiac time intervals for Mod-MPI calculation remains another key barrier to the development of a universal reference range to replace the various gestation-adjusted normal ranges published to date. There is an inherent subjectivity in manual selection of time points for measurement of cardiac time intervals, as reflected by ICCs of 0.8-0.9 when intra- and interobserver variability of Mod-MPI calculation is studied, even when calculation is performed by experienced operators using stringent, predefined criteria [47, 54]. With machine settings now having been optimised, we feel that the automated measurement of the Mod-MPI is the crucial next step towards the development of the Mod-MPI as a routinely utilised clinical tool.

We have developed a novel automated MPI system in a collaboration between fetal medicine practitioners and biomedical engineers [57]. The automation algorithm automatically locates valve click peaks and calculates the Mod-MPI using the morphological characteristics of the aortic and mitral flow in the Doppler ultrasound waveform and the intensity signals (or “brightness”) from waveform images [57]. Repeatability of the fetal left Mod-MPI when obtained using this automated system for fetal Mod-MPI measurement and a comparison with manual measurement have been the focus of recent research. Preliminary data suggest that Mod-MPI automation is sensitive, precise, and feasible when compared to an experienced human observer's acquisition of Mod-MPI values [57]. Our automation system is in the process of being internationally tested with the intention of ultimately open sourcing the algorithm.

Lee et al. have also proposed a system of automatically measuring the Mod-MPI [58]. Their Auto Mod-MPI system (Samsung Electronics Co. Ltd., Suwon, South Korea) detects valve clicks using a methodology which first requires the operator to manually select a region of interest in the Doppler waveform before any further image analysis can take place [59]. In comparison, our automated MPI system does not require manual selection of a region of interest, instead it analyses the entire image automatically. The valve clicks in our automated MPI system are found by a weighted sum of various signals in the region surrounding the valve click. Whilst both automation systems use the morphology of the waveform to identify the clicks, our system also uses intensity information of the image for identification of all four valve clicks. This is only possible for two of the four clicks in the automation system proposed by Yoon et al. [59]. Further, our automation system localises valve clicks independently of each other rather than sequentially [60].

Multiple groups have found that the IRT is the cardiac time interval for which repeatability has been demonstrated to be the poorest [54, 56] which is important because the IRT is often the first time interval to be affected in cardiac dysfunction [10]. The morphological characteristics of the mitral valve opening include lack of a reflected click and tendency for the valve opening to be thick and tilted [47]. This is an additional reason why the subjectivity in operator-dependent calliper placement is likely to be the major source of measurement imprecision and contributor to interoperator measurement variability, especially for the IRT but to a lesser extent the other time intervals. Automation of the Mod-MPI may facilitate the incorporation of the MPI as a routine measure of fetal cardiac function by removing the significant subjective component of manual calliper placement for measurement of the constituent time intervals.

Further, manual calculation is time-consuming and requires highly trained staff. An average of 65 fetal MPI measurements is required to attain competence at producing reliable measurements [61]. Automated calculation of the Mod-MPI is likely to significantly reduce the amount of time needed for a Mod-MPI value to be generated and facilitate Mod-MPI signal capture amongst sonographers with limited expertise in Mod-MPI acquisition [57]. If automated Mod-MPI proves to be readily applicable to the clinical setting, the subsequently generated automated Mod-MPI normal values will provide a universal reference range to replace the multiple prior manually derived ranges. This in turn will allow definitive assessment of whether differences in Mod-MPI values in pathological subgroups versus uncomplicated controls are sufficient for Mod-MPI to translate from research tool to clinical utility.

## 7. A Note on the Right Heart MPI

Progressive refinement of left Mod-MPI measurement has been facilitated by the fact that only a single waveform is required [1, 5, 48]. The Mod-MPI for the right heart has traditionally been a slightly more cumbersome measure. This is because the tricuspid and pulmonary valves are located in differing anatomical planes so that two waveforms from two different planes have been necessary for right Mod-MPI calculation, and the waveforms have not been obtainable from the same cardiac cycle [5]. Hernandez-Andrade et al. suggested that small potential variations in the fetal heart rate between recordings of the two waveforms could affect the reproducibility of the right Mod-MPI, although the degree of heart rate variation sufficient to impact reproducibility is uncertain. This concern regarding reproducibility may have contributed to most MPI research focusing on the left ventricle rather than the right in normal and pathological pregnancies following the introduction of the Mod-MPI [26, 40, 48, 62].

However, Meriki et al. have since demonstrated similar reproducibility of left and right MPI [54]. The right MPI is considered an important parameter because the fetus is right-heart dominant [63]. Furthermore, the right MPI may provide an earlier indication of the development of pathology because changes in right heart function precede changes in left heart function in the context of pathology such as TTTS [54]. Recently we have found that the right MPI can be acquired using a single plane, single waveform technique in fetuses until approximately 26 weeks of gestation when the tricuspid and pulmonary valves diverge (unpublished data). Thus earlier in pregnancy, a fetal right Mod-MPI may be derivable from a waveform identical to that which is currently used for left Mod-MPI calculation. This will allow an automated process to calculate the right Mod-MPI in the same way as for the left, thereby increasing the ease of clinical application and utility of the right Mod-MPI.

## 8. Conclusion

The MPI is a noninvasive marker of global myocardial function and a sensitive tool for detecting fetal cardiac dysfunction. Its translational potential is now mainly constrained by the lack of standardised methodology for demarcation of the time periods used in its calculation and the need for improvement of its repeatability and ease of measurement. Since the introduction of the Mod-MPI, various studies have been necessary to evaluate technical refinements in order to standardise its measurement. Automation of the measurement process may be considered as the next logical step towards unification of the Mod-MPI measurement process, and we hope towards its role as a cornerstone of functional fetal echocardiography.

## Figures and Tables

**Table 1:** Technique and machine settings used in various studies (adapted from Mahajan et al., in press [46]).

	Angle of insonation (°)	WMF (Hz)	Sample volume (mm)	Sweep velocity (cm/s)	Doppler gain	Placement of time cursor
Tei, 1995 [8]	ND	ND	ND	10	ND	Beginning of mitral inflow and aortic outflow waveforms.
Hernandez-Andrade et al., 2005 [5]	<30	70	3	15	Min.	Beginning of valve clicks.
Van Mieghem et al., 2009 [49]	<15	≥120	ND	10	ND	End of closing clicks to beginning of opening clicks.
Meriki et al., 2012 [48]	<15	300	3	15	Min.	Peak of valve clicks.
Lobmaier et al., 2014 for Siemens Antares machine^*^ [56]	<15	281	4	15	60 dB	Beginning of valve clicks.
Lobmaier et al., 2014 for Voluson 730 Expert machine^*^ [56]	<15	210	4	15	−10 dB	Beginning of valve clicks.

WMF: wall motion filter; ND: not defined; min.: minimum.

^*^Values displayed are the “optimal settings” for each ultrasound machine trialled in this study, as various settings were compared.
